# Non-endometrioid and high-grade endometrioid endometrial cancers show DNA fragmentation factor 40 (DFF40) and B-cell lymphoma 2 protein (BCL2) underexpression, which predicts disease-free and overall survival, but not DNA fragmentation factor 45 (DFF45) underexpression

**DOI:** 10.1186/s12885-018-4333-6

**Published:** 2018-04-13

**Authors:** Tomasz Banas, Kazimierz Pitynski, Krzysztof Okon, Aleksandra Winiarska

**Affiliations:** 1 Department of Gynecology and Oncology, Jagiellonian University, 21 Kopernika Street, 30-501 Krakow, Poland; 2 Department of Pathomorphology, Jagiellonian University Medical College, Krakow, Poland

**Keywords:** B-cell lymphoma 2 (BCL2), DNA fragmentation factor 40 (DFF40), DNA fragmentation factor 45 (DFF45), Endometrial cancers (ECs), Disease-free survival (DFS), Overall survival (OS)

## Abstract

**Background:**

The expression of DNA fragmentation factor 45 (DFF45) and B-cell lymphoma 2 (BCL2) in glands of the normal human endometrium is related to phases of the menstrual cycle and decreases after menopause, whereas the expression of DNA fragmentation factor 40 (DFF40) is stable. Moreover, DF45, BCL2 and DFF40 underexpression has been reported in numerous malignancies, including uterine leiomyosarcomas. In this study, we aimed to investigate DFF45, BCL2 and DFF40 expression in endometrioid and non-endometrioid types of endometrial cancers (ECs). We also evaluated the correlations between DFF45, BCL2 and DFF40 expression levels and clinicopathological parameters and determined the value of these three proteins as prognostic markers of disease-free survival (DFS) and overall survival (OS).

**Methods:**

Immunohistochemistry was performed to evaluate DFF45, BCL2 and DFF40 expression in 342 cases of ECs. Student’s t-test, the Mann-Whitney U-test, and the chi-squared test were used for the statistical analyses as appropriate. The Cox-Mantel test, Cox’s proportional hazard model, and relative risk analyses were used to evaluate associations between DFF40, DFF45, and BCL2 expression and clinicopathological characteristics.

**Results:**

DFF40 and BCL2, but not DFF45, were significantly underexpressed in non-endometrioid and high-grade endometrioid ECs compared with low- and moderate-grade endometrioid ECs. Women with DFF40- and BCL2-negative tumors had higher risks of disease recurrence, lymph node involvement, lympho-vascular space infiltration, and deep myometrial invasion compared with women with DFF40- and BCL2-positive tumors. Additionally, women with DFF40- and BCL2-negative tumors had significantly lower OS and DFS than women with DFF40- and BCL2-positive tumors. A multivariable analysis of the model, including the clinicopathological characteristics and immunohistochemical results, showed that negative BCL2 expression, lymph node involvement, and high-stage and high-grade disease were independent predictors of OS, whereas negative BCL2 expression, lymph node involvement, and high-stage disease were independent predictors of DFS.

**Conclusions:**

Compared with low- and moderate-grade endometrioid ECs, non-endometrioid and high-grade endometrioid ECs showed significant DFF40 and BCL2 underexpression. The absence of DFF40 and BCL2 expression negatively affects DFS and OS. Further prospective studies are warranted to assess the potential utility of DFF40 and BCL2 as targets in the diagnosis or treatment of ECs.

## Background

Endometrial cancers (ECs) have the highest incidence among female genital tract malignancies in economically prosperous populations [1, 2]. Epithelial malignant tumors of the uterus include pure endometroid cancers grade 1-3, uterine serous carcinomas, clear cell carcinomas, and carcinosarcomas [3]. According to their clinical features, Bokhman divided ECs into two groups: estrogen-dependent type 1 malignancies, which typically occur during the perimenopausal period and have a favorable prognosis, and estrogen-independent type 2 malignancies, the incidence of which is highest among individuals 70 years of age with a poor prognosis [4]. Subsequent studies have confirmed that type 1 and type 2 ECs differ with respect to their morphologies and molecular characteristics. Pure endometrioid ECs are histologically heterogeneous and present different patterns of myometrial invasion [5]. To describe an unusual type of this invasion, Murra et al. introduced the acronym “MELF” for microcystic, elongated and fragmentated pattern of myometrial invasion in endometrioid ECs, but they failed to demonstrate a statistically significant effect of MELF on overall survival – only a subgroup of women showing a fibromyxoid stromal reaction and MELF had improved survival [6]. Further studies have revealed that the incidence of MELF in ECs is between 13% and 36% and have shown that its presence is significantly associated with tumor size, lymph node involvement, advanced International Federation of Gynecology and Obstetrics (FIGO) stage, lympho-vascular space involvement (LVSI+), mucinosus differentiation, and papillary architecture [7–10]. In contrast to the findings reported by Murray et al. and Kihara et al., who did not demonstrate any association between MELF and disease-free or overall survivals, Zinkov et al. observed a significantly lower survival rate in patients with endometrioid ECs with a MELF pattern compared with MELF-negative women [6, 7, 11].

Type 2 malignancies are characterized by p53 and PIK3CA overexpression, and type 1 malignancies are characterized by PTEN mutations [12]. High-grade early-stage ECs display SOX-2 overexpression and low gamma-glutamyltransferase expression but not Oct4 overexpression. SOX-2 and Oct4 are cancer stem cell markers that have been hypothesized to be responsible for carcinoma infiltration, and gamma-glutamyltransferase acts as an antioxidant and thus protects cells against oxidative stress [13, 14]. Type 2 ECs also display increased proliferation and apoptosis rates [15].

Burstman was the first to investigate the role of DNA fragmentation factor 45 (DFF45) in ECs [16]. The DNA fragmentation factor (DFF) complex is localized to the cell nucleus and exists as a dimer comprising DFF40 (caspase-activated DNase; CAD) and DFF45 (inhibitor of caspase-activated DNase; ICAD) [17]. DFF45 is cleaved by caspase-9 at the end of the apoptotic cascade, which results in the release of DFF40 from the DFF40/DFF45 complex, leading to DFF40-mediated DNA fragmentation (DNA laddering) [10]. However, DFF45 is not merely an inhibitor of DFF40, as its presence is also required for the proper folding of DFF40, which is necessary for production of the active protein [17, 18]. In our recent studies, we have demonstrated DFF40 and DFF45 overexpression in endometrial polyps and benign endometrial hyperplasia compared with control endometrium [19]. Moreover, we have shown that uterine leiomyosarcomas are characterized by DFF40 and DFF45 underexpression compared with case and control myometrium; women whose tumors were negative for DFF40 presented significantly shorter disease-free survival and overall survival [20].

BCL2 is a well-known inhibitor of apoptosis that is responsible for stabilizing mitochondrial membranes and thus preventing cells from undergoing apoptosis via the mitochondrial pathway [21, 22]. BCL2 underexpression has been linked to more aggressive ECs, including high-grade cancers, advanced stage cancers, and cancers displaying lymph node invasion [23]. In contrast to benign endometrial polyps, significant BCL2 underexpression has been shown in the stromal layer of benign hyperplastic endometrium [18].

The aim of this work was to evaluate the relationship between the immunohistochemical expression patterns of DFF45 and DFF40 and those of BCL2 and to assess the correlations between the expression of these biomarkers and the clinicopathological characteristics of ECs.

## Methods

### Patient and tumor characteristics

This study was performed using archived paraffin-embedded tissue samples from patients who were diagnosed with and treated for ECs in the Gynecology and Oncology Clinic of Jagiellonian University, Krakow, Poland. Samples from 365 patients who were consecutively diagnosed with EC from January 2007 to December 2012 were initially considered eligible for inclusion in the study; however, the medical records for 24 of these women were found to have missing data, and the samples from an additional 17 patients underwent autolysis. Thus, samples from 342 patients were ultimately eligible for inclusion in the study. Each patient was preoperatively diagnosed with EC based on the results of endometrial evaluations performed via dilatation and curettage (D&C) or hysteroscopy with biopsy. Each patient also underwent pelvic ultrasound or pelvic magnetic resonance imaging, abdominal ultrasound, and chest X-ray or chest computed tomography preoperatively. Each patient subsequently underwent total hysterectomy with bilateral salpingo-oophorectomy with or without pelvic lymph node biopsy, and patients with an increased risk of disease recurrence and/or nodal metastases underwent radical surgery and/or pelvic/para-aortal lymph node dissection. The omentum was removed in patients with tumors with histology corresponding to serous carcinoma, clear cell carcinoma, or carcinosarcoma. Each postoperative specimen was re-evaluated by two experienced pathologists, who were blinded to each other’s interpretations and to previous pathology reports, to confirm the final diagnosis of EC. All cases were restaged using current FIGO 2010 criteria [3]. Moreover, histological typing, grading (G), and the presence of MELF pattern myometrial invasion in the patients’ paraffin-embedded tissue specimens were performed according to the classification of the World Health Organization (WHO) and criteria presented by Murray et al. [6, 24] Body mass index (BMI) was calculated by dividing body mass, as determined by preoperative measurements of standardized morning weights, by the square of the body height (kg/m^2^), as determined by preoperative measurements of height. The date of menopause onset was defined as the date of the final menstrual period, i.e., the date after which no menses were reported for a subsequent period of 12 months. Sub-cohorts comprising patients with G1-2 endometrial adenocarcinoma (Group 1) and patients with non-endometrioid EC and G3 endometrial adenocarcinoma (Group 2) were subsequently organized. The characteristics of these patients and their tumors are presented in Table 1.

**Table 1:** Baseline clinicopathological characteristics of studied endometrial cancers cohorts

	Entire Cohort *N* = 342	Group A *N* = 215	Group B *N* = 127	*p* ^*^
Histology	Endometrioid and non-endometrioid endometrial cancers	Low- and moderate- grade endometroid endometrial cancer	Non-endometrioid and high-grade endometroid endometrial cancer	
Age [years] (mean ± SD^**^)	67.424 ± 8.399	65.167 ± 6.661	71.244 ± 9.599	< 0.0001^#^
BMI [kg/m^2^] (mean ± SD^**^)	24.945 ± 3.195	25.774 ± 3.488	23.542 ± 1.952	< 0.0001^#^
Stage^***^				< 0.0001^#^
1	174 (50.88%)	133 (61.86%)	41 (32.28%)	
2	93 (27.19%)	45 (20.93%)	48 (37.80%)	
3	71 (20.76%)	35 (16.28%)	36 (28.35%)	
4	4 (1.17%)	2 (0.93%)	2 (1.57%)	
Grade				< 0.0001^#^
1	90 (26.31%)	90 (41.86%)	0 (0.00%)	
2	132 (38.60%)	125 (58.14%)	7 (5.51%)	
3	120 (35.09%)	0 (0.00%)	120 (94.49%)	
MI^***^ ≥ 50%	258 (75.44%)	148 (68.84%)	110 (86.61%)	0.0002^#^
+LN^&^	40 (11.70%)	15 (7.44%)	25 (19.68%)	0.0004^#^
+LVSI^&&^	94 (27.49%)	42 (19.53%)	52 (40.94%)	< 0.0001^#^
+MELF^&&&^	18 (5.26%)	18 (8.37%)	0 (0.00%)	< 0.0001^#^
Adjuvant therapy				< 0.0001^#^
VBTH^A^	175 (51.17%)	107 (49.77%)	68 (53.54%)	
ERTH^B^	26 (7.61%)	0 (0.00%)	26 (20.47%)	
CHT^C^	33 9.65%)	32 (14.88%)	1 (0.79%)	
HTH^D^	2 (0.58%)	2 (0.92%)	0 (0.00%)	
VBTH^A^ + ERTH^B^	10 (2.92%	0 (0.00%)	10 (7.87%)	
ERTH^B^ + CHT^C^	25 (7.31%)	3 (1.39%)	22 (17,33%)	
none	71 (20.76%)	71 (33.04%)	0 (0.00%)	
Recurrence	46 (13.45%)	20 (9.30%)	26 (20.47%)	0.0034^#^

* Comparison between Groups A and B; *SD – standard deviation; *** stage of disease according to the 2010 (FIGO) classification; ***MI - myometrial invasion; ^&^LN - lymph node involvement; ^&&^LVSI - lymphovascular space invasion; ^#^statistically significant p-value; ^A^VBTH – vaginal brachytherapy; ^B^ERTH – external radiotherapy; ^C^CHT – chemotherapy; ^D^HTH – hormonal therapy

### Sample preparation

Representative postoperative tissue blocks were identified for immunohistochemical analysis. Four specimens from four patients with stage 4 disease that were obtained by D&C were also included in the analysis. Immunohistochemical staining was performed with a rabbit polyclonal antibody against DFF40 (Abcam, Cambridge, UK) diluted 1:50, a monoclonal mouse anti-human antibody against BCL2 (Leica Biosystems Newcastle Ltd., Newcastle Upon Tyne, UK) diluted 1:200 and a rabbit polyclonal antibody against DFF45 (Abcam, Cambridge, UK) diluted 1:100 using a previously described protocol [19, 20].

Sections that were stained but incubated without a primary antibody were used as negative controls. Jurkat cells (for DFF45 and DFF40) and human follicular lymphoma cells (for BCL2) were used as positive controls.

### Immunohistochemical scoring

To obtain comparable outcomes to our formerly published results, the following procedure, which was previously developed and validated, was employed: two certified histopathologists, who were blinded to the study data and to each other, calculated the staining scores for DFF40, BCL2 and DFF45 for each slide in five high-power fields (× 40) using a 0-to-3 scale (0, no staining; 1, weak staining; 2, moderate staining; and 3, strong staining) [19, 20]. The cell staining percentage scores for DFF45 and DFF40 were determined as follows: 0 = expression in up to 10% of cells; 1+ = expression in 10–50% of cells; 2+ = expression in 51–80% of cells; and 3+ = expression in over 80% of cells. The cell staining percentage scores for BCL2 were calculated using the scale defined by Yigit et al.: 0 = expression in up to 5% of cells; 1+ = expression in 5%-25% of cells; 2+ = expression in 26%-50% of cells; and 3+ expression in over 50% of cells [25]. The intensity score was multiplied by the cell staining percentage score to obtain the final immunoreactivity score for each protein, which ranged from 0 to 9. Final scores of 0-1 were indicative of negative protein expression, whereas scores of 2-4 were indicative of low protein expression, and scores of 6-9 were indicative of high protein expression. Discrepancies regarding scoring were noted in 1.75% of the cases. In these instances, the corresponding samples were re-evaluated 2 weeks after the primary evaluation to achieve consensus regarding their scores and to minimize the possibility that the results of analysis would be affected by recall bias.

### Statistical analysis

The clinical characteristics of the study groups were compared using the parametric Student’s t-test and the non-parametric Mann–Whitney U test as appropriate. The data are presented as the mean ± standard deviation (SD) or as the median ± standard error of the mean (SEM). The chi-squared test was employed to evaluate differences in biomarker immunoexpression, with Yate’s correction, if required, based on the expected frequencies of variables. Cox’s proportional hazard model was used to determine the hazard ratios (HRs) and corresponding 95% confidence intervals (CIs) for the survival predictors. The Cox–Mantel test was used to compare OS (defined as the period between the initial surgery and time of death) and DFS (defined as the period between the initial surgery and time of disease recurrence), and the relative risks (RRs) of the factors associated with a poor prognosis were calculated for cases in which DFF40, DFF45, or BCL2 expression was negative. To evaluate interobserver/intraobserver agreement for the immunohistochemistry scores, we calculated the intraclass correlation coefficient (ICC) and corresponding 95% CIs for multiple histopathological evaluations. For these evaluations, Research Randomizer (www.randomizer.org) was employed to select 50 samples in which DFF40, DFF45, and BCL2 expression levels were re-evaluated at 2 weeks after the primary evaluation to prevent recall bias from affecting the study results. The Guidelines for Reporting Reliability and Agreement in Studies were used to verify these results [26]. Calculations were performed using STATISTICA data analysis software, version 12.0 (StatSoft, Inc. 2014. STATISTICA data analysis software system, version 12. www.statsoft.pl), and MedCalc Statistical Software, version 16.2.1 (MedCalc Software by Ostend, Belgium). A *p* value of 0.05 was considered statistically significant.

## Results

### Clinical characteristics

A total of 342 patients were included in analysis. The median follow-up duration was 64 (IQR: 42.00; range: 11-106) months. Multivariate analysis showed that stage 3+ disease (HR: 4.811; CI: 2391-9.681; *p* < 0.001), G3 disease (HR: 3.040; CI: 1.474-6.2711; *p* = 0.003), lymph node metastases (LN+) (HR: 2.834; CI:1.447-3.730; *p* = 0.002), and LVSI+ (HR: 1.976; CI: 1.047-3.730; *p* = 0.036) were significant predictors of OS, while age, BMI, positive peritoneal washings, and adjuvant therapy were not independent predictors of OS. Multivariate analysis also showed that stage 3+ disease (HR: 3.354; CI: 1.643-6.846; *p* < 0.001), LN+ (HR: 3.561; CI: 1.831-6.924; *p* = 0.001), and LVSI+ (HR: 2.068; CI: 1.108-3.862; *p* = 0.023), but not G3, age, BMI, positive peritoneal washings, or adjuvant therapy, were independent predictors of DFS. The clinicopathological characteristics of the study cohort and its sub-groups are shown in Table 1.

### DFF40, DFF45, and BCL2 immunohistochemical expression

**Fig. 1:**
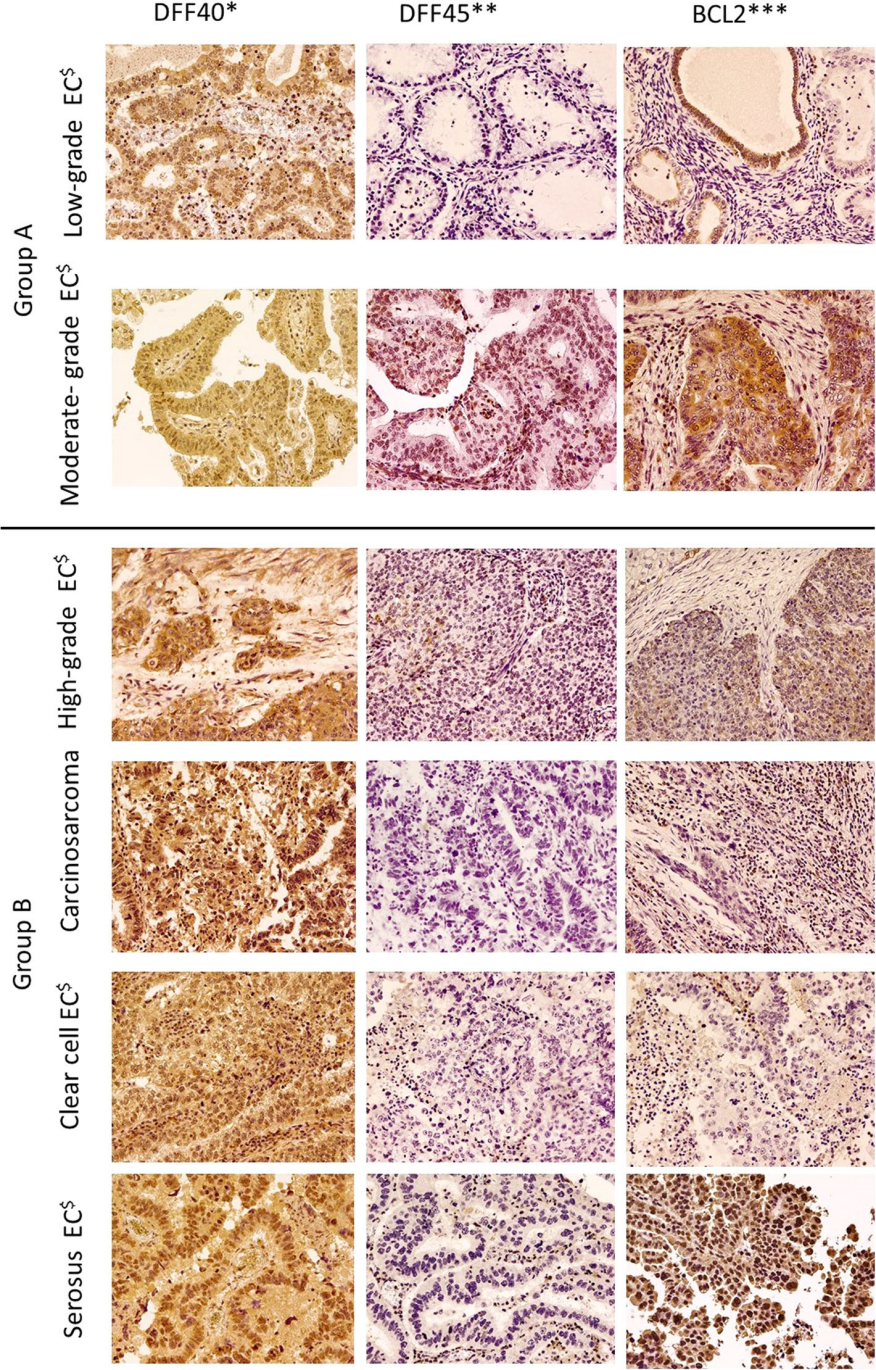
DFF40*, DFF45**, and BCL2*** expression (× 200) in different histological types of endometrial cancers. *DNA fragmentation factor 40; **DNA fragmentation factor 45; ***B-cell lymphoma 2 protein; ^$^Endometrial cancer

DFF40 and DFF45 displayed predominately nuclear expression, while BCL2 displayed cytoplasmic expression in all histological types of EC analyzed herein (Fig. 1). No differences in DFF40, DFF45, or BCL2 expression were observed between G1 ECs and G2 ECs in Group A, and no differences in DFF40, DFF45, and BCL2 expression were observed between G3 ECs and non-endometrioid tumors in Group B (Table 2). However, DFF40 and BCL2 expression levels were significantly higher in Group A than in Group B (*p* < 0.001 and p < 0.001; respectively). DFF45 expression levels did not differ between Groups A and B (Table 2).

**Table 2:** Immunoexpression of DFF40^*^, DFF45^**^, and BCL2^***^ in endometrial cancers in relation to histological type and study groups A and B

	Low and moderate-grade endometroid cancer (Group A)		p^A^	Non-endometrioid and high-grade endometroid endometrial cancer (Group B)				p^B^
Tumor histology	EAC-G1^$^ *N* = 90	EAC-G2^$$^ *N* = 125		Carcinosarcomas *N* = 31	Serosus tumor *N* = 22	Clear cell tuor *N* = 25	EAC-G3^$$$^ *N* = 49	
DFF40^*^								
High	31 (34.44%)	28 (22.40%)	0.143	5 (16.13%)	2 (9.09%)	1 (4.00%)	4 (8.16%)	0.754
Low	33 (36.67%)	52 (41.60%)		6 (19.35%)	3 (13.64%)	6 (24.00%)	11 (22.45%)	
Negative	26 (28.89%)	45 (36.00%)		20 (64.52%)	17 (77.27%)	18 (72.00%)	34 (69.39%)	
*p*^AB^ < 0.001^#^								
DFF45^**^								
High	1 (1.11%)	0 (0.00%)	0.409	1 (3.22%)	0 (0.00%)	0 (0.00%)	2 (4.08%)	0.935
Low	7 (7.78%)	13 (10.40%)		3 (9.68%)	2 (9.09%)	3 (12.00%)	10 (20.41%)	
Negative	82 (9.11%)	112 (89.6%)		27 (87.10%)	20 (90.91%)	22 (88.00%)	37 (75.51%)	
*p*^AB^ = 0.101								
Bcl-2^***^								
High	29 (32.22%)	44 (35.20%)	0.523	2 (6.45%)	1 (4.55%)	1 (4.00%)	5 (10.21%)	0.187
				4 (12.90%)	4 (18.18%)	3 (12.00%)		
Low	34 (37.78%)	38 (30.40%)					16 (32.65%)	
Negative	27 (30.00%)	43 (34.40%)		25 (80.65%)	17 (77.27%)	21 (84.00%)	28 (57.14%)	
*p*^AB^ < 0.001^#^								

p^A^ – chi-squared test in Group A; *p*^B^ – chi-squared test in Group B; *p*^AB^ – chi-squared test between Group A and Group B; ^$^endometrial adenocarcinoma grade 1; ^$$^endometrial adenocarcinoma grade 2; ^$$$^endometrial adenocarcinoma grade 3; ^*^DFF40 – DNA fragmentation factor 40; ^**^DFF45 – DNA fragmentation factor 45; ^***^BCL2 – B-cell lymphoma 2

The absence of DFF40 expression significantly increased the RRs for disease recurrence, LN+, LVSI+, and invasion of more than 50% of the myometrium (MI > 50%) in the entire cohort and in Group B (with the exception of the RR for MI > 50% in Group B) (Table 3). Similarly, BCL2 deficiency increased the RRs for disease recurrence, LN+, LVSI+, and MI > 50% in the entire cohort and in Groups A (with the exception of the RR for disease recurrence in Group A) and B (with the exception of the RR for MI > 50% in Group B). The absence of DFF45 expression was not associated with increases in the risks of any of the above factors, either in the entire cohort or in Group A or B (Table 3).

**Table 3:** Relative risks (RRs) and 95% confidence intervals (CIs) of recurrence (R+), lymph node involvement (LN+), lymphovascular space invasion (LVSI+), and myometrial invasion over 50% (MI > 50%) in patients with DFF40*-, DFF45**-, and BCL2***-negative endometrial cancers

	Low and moderate-grade endometroid cancer (Group A)	Non-endometrioid and high-grade endometroid endometrial cancer (Group B)	Total cohort
R+			
DFF40*	1.611 (CI:0.694-3.741; *p* = 0.267)	7.423 (CI: 1.048-52.599; *p* = 0.046)	2.435 (CI: 1.365-4.343; *p* = 0.002)
DFF45**	0.892 (CI:0.222-3.581; *p* = 0.871)	1.843 (CI: 0.608-5.586; *p* = 0.280)	1.211 (CI:0.506-2.898; *p* = 0.668)
BCL-2***	2.048 (CI: 0.821-5.081; *p* = 0.121)	12.847 (CI: 1.805-91.464; *p* = 0.012)	4.192 (CI: 2.128-8.261; *p* < 0.001)
LN+			
DFF40*	1.033 (CI:0.567-1.766; *p* = 0.915)	7.422 (CI:1.048-52.594; *p* = 0.045)	4.19 (CI: 1.88-9.33; *p* < 0.001)
DFF45**	0.433 (CI:0.133-1.412; *p* = 0.166)	1.161 (CI: 0.441-3.060; *p* = 0.763)	0.70 (CI: 0.33-1.48; *p* = 0.344)
BCL-2***	3.386 (CI:1.256-9.127; *p* = 0.015)	3.624 (CI:1.493-11.424; p-0.028)	4.36 (CI: 2.14-8.87; *p* < 0.001)
LVSI+			
DFF40*	0.947 (CI: 0.508-1.767; *p* = 0.865)	2907 (CI: 1.273-6.641; *p* = 0.011)	1.953 (CI:1.360-2.803;*p* < 0.001)
DFF45**	0.844 (CI: 0.374-1.907; *p* = 0.684)	1.736 (CI: 0.846-3.563; *p* = 0.133)	1.378 (CI: 0.749-2.537; *p* = 0.303)
BCL-2***	2.483 (CI: 1.459-4.226; *p* < 0.001)	3.624 (CI:1.493-11.424; p-0.028)	3.089 (CI:2.075-4.599; *p* < 0.001)
MI > 50%			
DFF40*	1.058 (CI: 0.873-1.283; *p* = 0.562)	1.183 (CI:0.962-1.456; *p* = 0.112)	1.216 (CI: 1.078-1.270; *p* = 0.001)
DFF45**	1.057 (CI: 0.668-1.672; *p* = 0.812)	1.189 (CI: 0.895-1.580; *p* = 0.233)	1.086 (CI: 0.888-1.328; *p* = 0.422)
BCL-2***	1.335 (CI: 1.332-1.574; *p* < 0.001)	1.073 (CI: 0.920-1.251; *p* = 0.369)	1.549 (CI: 1.346-1.782; *p* < 0.001)

*DFF40 - DNA fragmentation factor 40; **DFF45 - DNA fragmentation factor 45; ***BCL2 - B-cell lymphoma 2

**Fig. 2:**
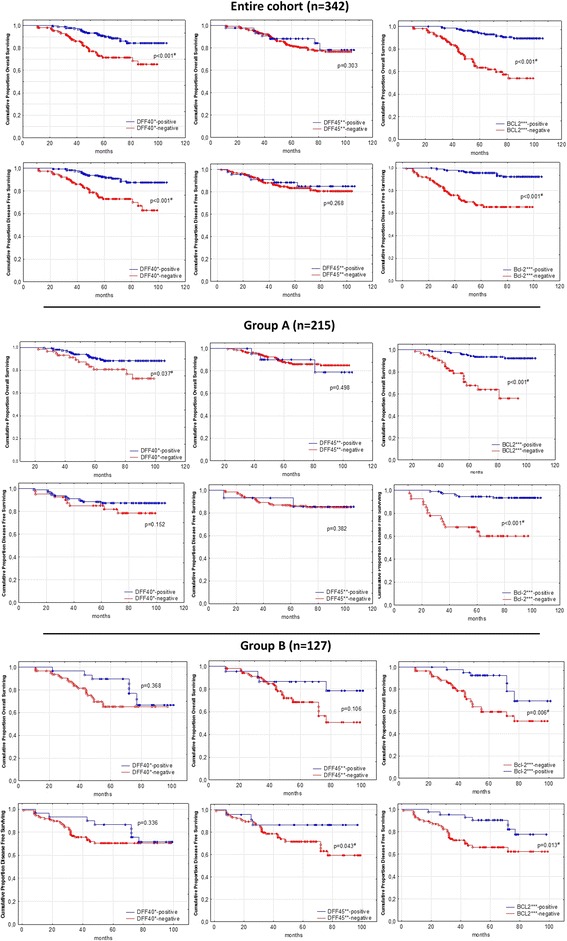
Kaplan-Meier survival curves for overall and disease-free survival in DFF40*-, DFF45**-, and BCL2***-negative and positive cases in the entire study cohort and in Groups **A**^$^ and **B**^$$^, respectively. *DFF40 - DNA fragmentation factor 40; **DFF45 - DNA fragmentation factor 45; ***BCL2 - B-cell lymphoma 2; ^$^Group **A** – includes low- and moderate-grade endometroid endometrial cancers; ^$$^Group **B** – includes non-endometroid and high-grade endometroid endometrial cancers; ^#^*p*-value statistically significant

DFF40-negative cases showed significantly shorter OS in the entire cohort and in Group A and displayed significantly shorter DFS in the entire cohort, but not in Group A or B, compared with DFF40-positive cases (Fig. 2). DFF45 expression did not affect OS or DFS in the entire cohort or in Group A; however, DFF45-negative patients in Group B exhibited shorter DFS, but not OS. BCL2-negative patients displayed significantly shorter OS in the entire cohort and in Groups A and B compared with BCL2-positive patients (Fig. 2). Similarly, BCL2-negative patients displayed significantly shorter DFS in the entire cohort and in Groups A and B compared with BCL2-positive patients (Fig. 2).

Univariate analysis revealed that decreased DFF40 and BCL2 expression (but not decreased DFF45 expression) increased the negative HRs for OS (HR: 2.757; CI: 1.644-4.624; *p* < 0.001 and HR: 6.277; CI: 3.522-11.189; p < 0.001; respectively) and DFS (HR: and DFS (HR: 2.937; CI: 1.680-5.134; p < 0.001 and HR: 6.979; CI: 3.654-13.331; p < 0.001; respectively) in the entire cohort. We subsequently performed multivariate analysis of the relationships among the clinical features and DFF40, DFF45, and BCL2 expression levels and OS and DFS in the entire cohort. The results showed that stage 3+ disease (HR: 3.605; CI: 1.763-7.372; *p* < 0.001), G3 disease (HR: 2.467; CI: 1.845-5.135; *p* = 0.016), LN+ (HR: 3.290; CI: 1.865-5.805; p < 0.001), and BCL2 negativity (HR: 3.105; CI: 1.654-5.827; p < 0.001), but not LVSI+, MI > 50%, age, positive peritoneal washings, adjuvant therapy, or DFF40 or DFF45 negativity, were significant predictors of OS. Additionally, stage 3+ (HR: 2.682; CI: 1.312-5.487; *p* = 0.007), LN+ (HR: 3.997; CI: 2.217-7205; p < 0.001), and BCL2 deficiency (HR: 2.302; CI: 2.302-8.777; p < 0.001) were significant predictors of DFS.

### Validation of the reliability of the immunohistochemistry scores

We noted very high intra-rater agreement with regard to the immunohistochemistry scores for DFF40, DFF45, and BCL2 expression. The ICCs for agreement between the immunohistochemistry scores for the indicated proteins, which were determined by the abovementioned pathologists, are listed below.

A) The ICCs for agreement between the scores for DFF40, DFF45, and BCL2 expression determined by the first pathologist (that is, observer 1 vs. observer 1 after 2 weeks) were 0.903 (CI: 0.875–0.968), 0.887 (CI: 0.823–0.986), and 0.919 (CI: 0.893–0.983), respectively.
B) The ICCs for agreement between the scores for DFF40, DFF45, and BCL2 expression determined by the second pathologist (that is, observer 2 vs. observer 2 after 2 weeks) were 0.934 (CI: 0.889–0.961), 0.898 (CI: 0.831–0.929), and 0.984 (CI: 0.972–0.990), respectively.

As full inter-rater consensus was achieved for the expression levels of the above proteins, we did not evaluate inter-observer reliability.

## Discussion

The study evaluates DFF45, BCL2 and DFF45 expression in ECs and assesses the relationships between DFF40 and clinical and histopathological factors. Although Buchman’s clinical classification of ECs into two groups has been validated in numerous molecular studies, it is currently being revised based on advances in cancer epidemiological research. Based on the results of a case-control study nested in the Women’s Health Imitative Observational Study, Suarez et al. and Brinton et al. have postulated that serous endometrial tumors behave in a manner similar to that of estrogen-dependent endometrial malignancies under some circumstances [27, 28]. Yang et al. have shown that diabetes also increases the risks of serous tumors and type I ECs, but not that of clear cell endometrial tumors, and that it increases the risk of type 1 ECs [29]. Regarding factors associated with cancer prevention, Sherman et al. have postulated that cigarette smoking, which is a well-known protective factor against type 1 ECs, also reduces the risk of serous endometrial tumors [30]. However, the results of our study confirmed Bokhman’s scheme, as they showed that DFF40 and BCL2 expression levels were decreased in non-endometrioid and high-grade endometrial tumors compared with grade 1 and 2 ECs. These results are consistent with those of Sakuragi et al., Morsi et al., Loffe et al., and Porichi et al., who reported decreased BCL2 expression levels in ECs with increased mitotic and apoptotic indices [31–34]. Lack of BCL2 expression was subsequently shown to be an independent predictor of poor prognosis in ECs, and high disease stage, high grade, increased invasion depth, and disease recurrence were shown to be independent prognostic factors for shorter OS [32, 35]. We showed that BCL2 deficiency increased the risk of lymph node involvement and LVSI+ in both endometrioid and non-endometrioid tumors. Additionally, we showed that BCL2 deficiency was associated with increased risks of recurrence and deep myometrial invasion in the entire cohort.

In contrast to the significance of BCL2 expression, the implications of DFF40 and DFF45 expression alterations in ECs have been poorly addressed. Burstmann was the only author to show that DFF45 immunoreactivity was increased in atypical endometrial hyperplasia compared with normal endometrial tissue and non-atypical endometrial hyperplasia, but he failed to demonstrate that DFF45 expression differed between atypical endometrial hyperplasia and ECs [16]. Moreover, he analyzed only 48 cases of endometrioid ECs and observed no association between DFF45 expression and EC stage or grade [16]. Our results are consistent with those of Burstmann because as we did not observe an association between EC type and DFF45 expression, and we did not observe an effect of DFF45 expression on the clinical features of tumors, OS, or DFS in the entire cohort or in Group A, In contrast, only patients in Group B with DFF45-negative expression showed lower DFS, but not OS. However, DFF45 upregulation has been observed in ovarian serous cancers and colon cancers, and high DFF45 expression has been associated with poorer OS in previous studies [36, 37]. In contrast, DFF45 expression has been reported to be significantly reduced in esophageal squamous cell cancers with higher stages and in those with lymph node involvement and extensive lymphovascular space invasion than in cancers with lower stages and without lymph node involvement or extensive lymphovascular space invasion; moreover, patients with low DFF45 expression levels have been demonstrated to have significantly shorter OS than those with high DFF45 expression levels [38].

DFF40 expression in endometrial malignancies was a novel issue addressed in our study because it has not been previously investigated, and this novelty precludes direct comparisons between our results and those of other studies. Therefore, a literature review regarding DFF40 expression in other malignancies was performed. In glioblastoma cells, Judson et al. failed to observe DFF40 mutations, whereas George et al. showed increased DFF40 levels during Taxol-mediated apoptosis in different human glioblastoma lines [39, 40]. A lack of oligonucleosomal DNA fragmentation in human renal cell carcinomas that are resistant to apoptosis was associated with decreased DFF40 and DFF45 expression [41]. Mizuta et al. proved that the presence of DFF40 is mandatory for DNA fragmentation during apoptosis in Burkitt cell lymphoma, and Lucieano et al. reported that DFF40 downregulation might be a mechanism through which cancer cells avoid apoptosis in this malignancy [42, 43]. Breast cancer cells with DFF40 overexpression are more sensitive to doxorubicin, acetazolamide, and sulfabenzamide [44, 45]. These results indicate that DFF40 participates in the late stages of apoptosis and that it can be downregulated in some malignancies. We observed that DFF40 downregulation was associated with lower OS and DFS in the entire cohort and in Group A. However, unlike BCL2 underexpression, DFF40 underexpression was not identified as an independent predictor of OS or DFS in our multivariate analysis.

The current study has some limitations that must be presented. We recognize that immunohistochemistry might be susceptible to observer bias; however, this technique has been generally accepted for measuring DFF45 and BCL2 expression in previous studies, and the perfect inter-rater agreement and almost perfect intra-rater correlation noted herein make observer bias an insignificant contributor to the final results [16, 19, 23, 32, 36, 46]. Furthermore, immunostaining does not enable researchers to measure DFF40:DFF45 nuclear stoichiometric ratios in EC cells. Widlak et al. reported that a 1:1 DFF40:DFF45 ratio is essential for proper apoptosis, but this parameter was not assessed in our study [47]. Although the MELF pattern of myometrial invasion has been reported to be associated with worse clinical findings, we were unable to analyze its association with DFF40, DFF45, and BCL2 and its impact on DFS and OS expression due to the small number of identified cases. Finally, the core strength of this study was its comprehensive analysis of DFF40 and DFF45 expression and BCL2 expression in ECs. As previous studies have reported discrepancies regarding the pathological classifications of different types of ECs, all the specimens included herein were independently re-evaluated by two experienced pathologists to confirm their diagnoses and classifications [48]. Additionally, the relatively large size of the cohort, as well as the well-defined clinical characteristics of the specimens included herein, allowed us to analyze the relationships between DFF40, DFF45, and BCL2 expression and the clinical characteristics, including OS and DFS.

## Conclusions

Non-endometrioid and high-grade endometrioid ECs show decreased expression of DFF45, BCL2 and DFF40 compared with low- and moderate-grade endometrioid ECs. Additionally, DFF40 and BCL2 expression, but not DFF45 expression, together with disease recurrence, lymph node involvement, lymphovascular space invasion, and deep myometrial infiltration might predict DFS and OS. An assessment of the relationships between DFF40, BCL2 and DFF45 expression and the above factors would enable a better understanding of the significance of DFF40, DFF45, and BCL2 expression in other malignancies, and prospective studies regarding the utility of DFF40 and BCL2 as prognostic factors in ECs are warranted.

## Abbreviations

BCL2: B-cell lymphoma 2
BMI: Body mass index
CHT: Chemotherapy
DFF40: DNA fragmentation factor 40
DFF45: DNA fragmentation factor 45
DFS: Disease-free survival
ECs: Endometrial cancers
ERTH: External radiotherapy
HTH: Hormonal therapy
LN: Lymph node Involvement
LVSI: Lymphovascular space invasion
MELF: Microcystic, elongated and fragmentated pattern of myometrial invasion
OS: Overall survival
VBTH: Vaginal brachytherapy
